# Malignant peripheral nerve sheath tumors of the sino-nasal tract: about an unusual case report

**DOI:** 10.1093/jscr/rjac028

**Published:** 2022-02-18

**Authors:** Ismail Boujida, Hafsa Elouazzani, Sabrine Derqaoui, Hicham Belghiti, Zahra Sayad, Malik Boulaades, Fouad Zouaidia, Nadia Cherradi

**Affiliations:** 1 Department of Pathology HSR, Ibn Sina University Hospital Center, Rabat 10100, Morocco; 2 Faculty of Medicine and Pharmacy, Mohammed 5 University, Rabat 10100, Morocco; 3 Department of Maxillofacial Surgery, Ibn Sina University Hospital Center, Rabat 10100, Morocco

## Abstract

Malignant peripheral nerve sheath tumors are defined as malignant tumors arising from or differentiating toward the cells of the peripheral nerve sheath. They occur in about 8–16% within the head and neck region. Morphologically, some malignant tumors look like malignant peripheral nerve sheath tumors, particularly in the head and neck location; however, immunohistochemistry have a great contribution to distinguish between them. This case report is on a 45-year-old woman with a malignant peripheral nerve sheath tumor located in the sino-nasal tract.

## INTRODUCTION

Malignant peripheral nerve sheath tumors (MPNSTs) are very rare sarcomas of the nasal cavity and paranasal sinuses. [1] It is defined as any malignant neoplasm arising from or differentiating toward the cells of the peripheral nerve sheath, apart for tumors originating from the epineurium or the peripheral nerve vasculature. [2] MPNSTs account for about 5–10% of all soft-tissue sarcomas, with only about 8–16% occurring within the head and neck region [3]. Correspondingly, a MPNST of the head and neck region is extremely rare and even rarer when located in the nasal cavity or paranasal sinuses. [1]

Herein we describe a case of MPNST located in the nasal cavity of a North African 45-year-old woman with a brief review of literature to highlight the challenging diagnosis in this incoming presentation.

## CASE

**Figure 1:**
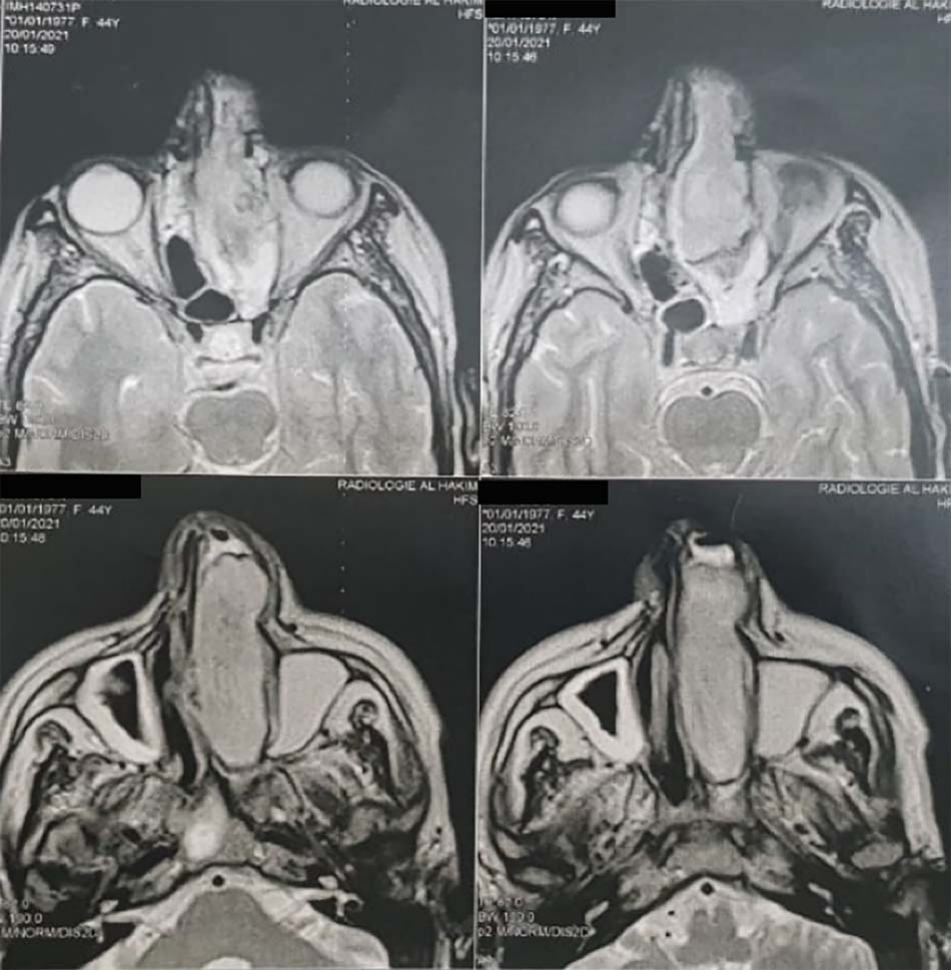
Head MRI showing a filling of the left nasal cavity by a neoplastic process.

A 45-year-old patient with no significant pathological history was admitted to the otorhinolaryngology department in March 2021. She had a 7 months history of purulent rhinorrhea, occasional epistaxis associated with swelling of the left nasal cavity gradually increasing in size. Clinical examination found a 6 cm whitish firm mass, filling the left nasal cavity, firm and painful on palpation and deflecting the dorsum and tip of the nose to the right. A facial magnetic resonance imaging (MRI) was performed showing a tissue process in the left nasal cavity (Fig. 1). An initial biopsy performed suggested a synovialosarcoma. The patient then underwent surgical resection preceded by two sessions of chemotherapy, allowing tumor size reduction.

**Figure 2:**
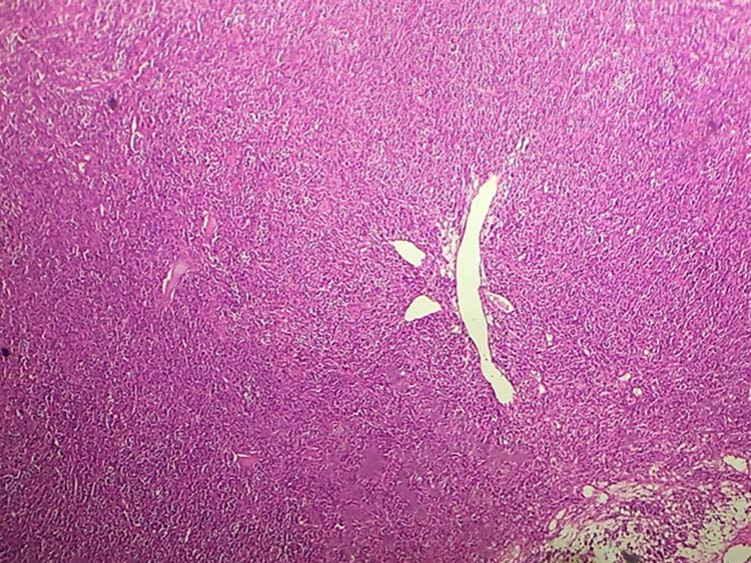
HE section showing diffuse fasciculated tumor (x10).

**Figure 3:**
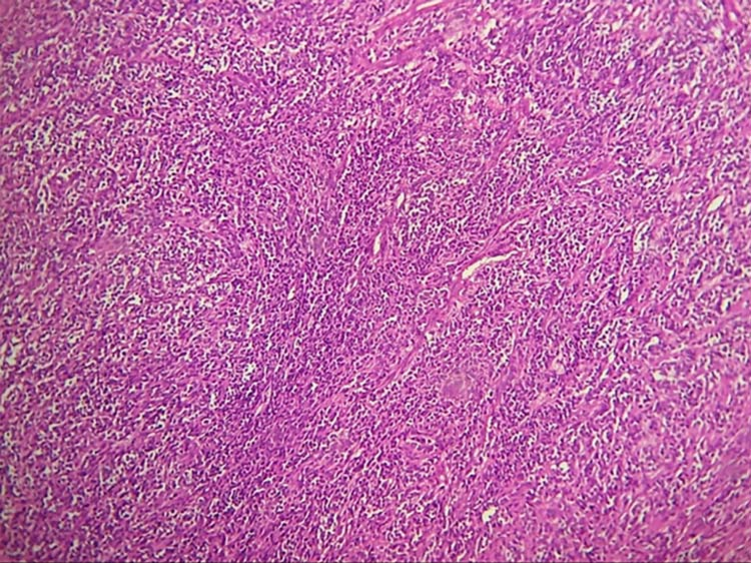
HE section showing diffuse fasciculated tumor (x20).

**Figure 4:**
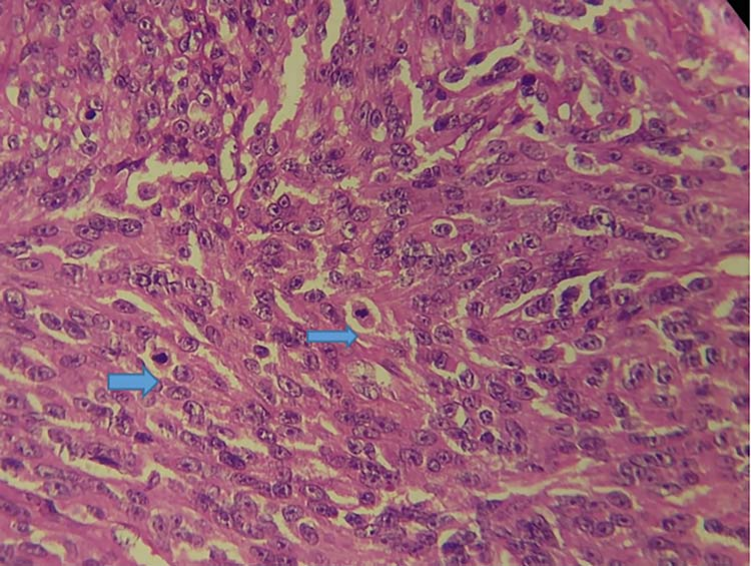
HE section showing tumor cells pleomorphisme and mitotic figures (blue arrow) (x40).

**Figure 5:**
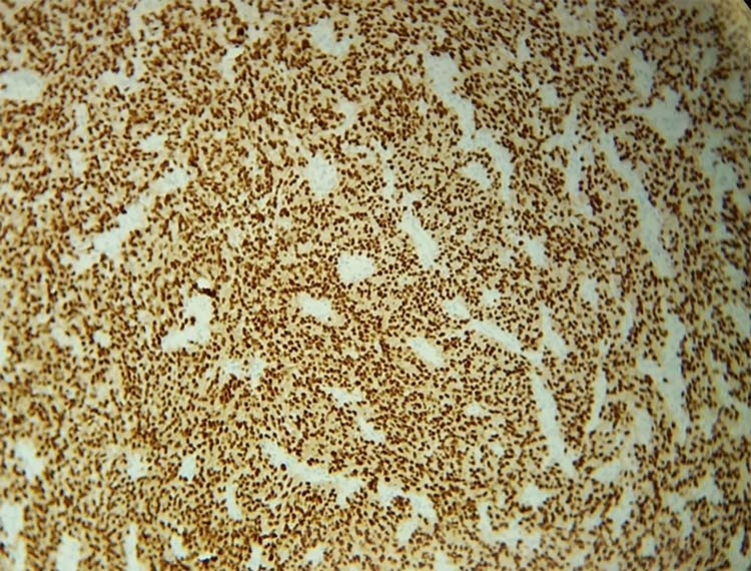
IHC: tumor cells express diffusely and intensely SOX10.

**Figure 6:**
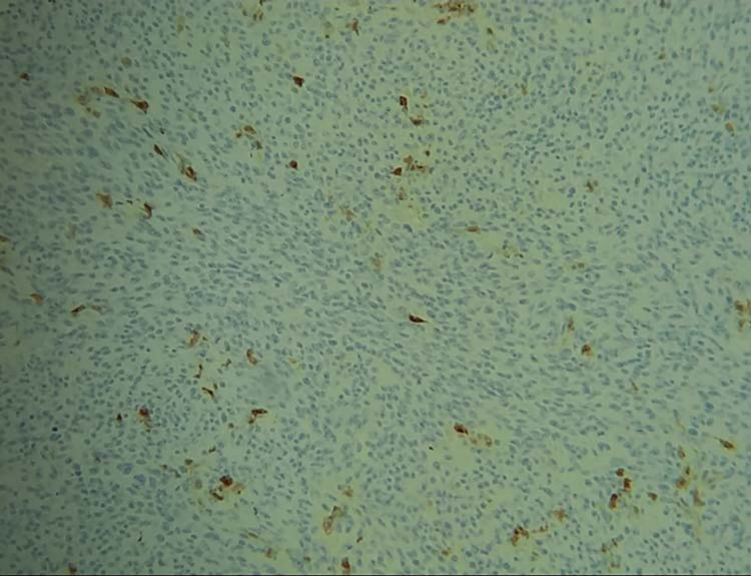
IHC: tumor cells express focally PS100.

Macroscopically, the tumor was whitish, fleshy, firm, with a polylobed surface and harboring foci of necrosis. Histological examination showed densely cellular spindle cell proliferation without evidence of differentiation, with focal nuclear pleomorphism and few foci of tumor necrosis (<50%). Mitotic activity was high, estimated at 13 mitoses/10 fields at high power. (Figs 2–4) An exhaustive immunohistochemical study was necessary to classify this tumor (Cytokeratin AE1/AE3, Melan A, Epithelial Membrane Antigen (EMA), PS100, Smooth Muscle Actin (SMA), H-Caldesmone, Desmin, Myogenin, CD34, CD31, Chromogranin, Synaptophysin, CD56, P63, Beta-Catenin, STAT6 and SOX10). Tumor cells expressed SOX10 intensely and diffusely (Fig. 5), and PS100 focal (Fig. 6). The other markers were not expressed and the Ki 67 proliferation index was estimated at 60%.

The diagnosis finally retained is that of MPNST of high grade (grade III of the FNCLCC).

The patient was referred to the oncology department for further treatment.

## DISCUSSION

MPNSTs are very rare tumors, and it has a very low incidence of about 0.001% within the population. These tumors are very infrequent in the head and neck, and they usually affect the proximal extremities and trunk [3]. They typically arise in adulthood, with most occurring in the third, fourth and fifth decade of age [4]. There are only few cases described in the literature.

About half of MPNSTs happen within the setting of hereditary syndrome neurofibromatosis type 1 (NF1), whereas 10% of MPNST are related to prior history of radiation. For the remaining cases, MPNST develops sporadically, without any previous radiation or known genetic predisposition. [5]

Clinically, MPNSTs generally appear as a progressively enlarging painless mass. Like the other sinus paranasales neoplasms, MPNSTs in this anatomic region may present clinically with unilateral nasal obstruction, epistaxis, hyposmia, hypoaesthesia, atypical pain or swelling of the nasal region, headache and mucopurulent rhinorrhoea. It may also give exophthalmos and impairment of a nerve. [6]

Malignant tumors of the peripheral nerve sheaths are lesions that can sometimes be responsible for expansion of the nerve from which they originate. Depending on the stroma and cellularity, tumors can be fleshy, fibrous or gelatinous. Macroscopically, MPNSTs are large masses, producing swelling of major nerves. Microscopically, most MPNSTs have a richly cellular fasciculated architecture made up of generally monomorphic spindle cells with eosinophilic cytoplasm of hyperchromatic rounded or oval nuclei and indistinct cell boundaries. There is often some degree of nuclear pleomorphism. Mitotic figures are more often found in intermediate and high-grade tumors and therefore more rarely in low-grade MPNST. Some cases of MPNST show high cellularity and diffuse throughout the tumor, with a pattern of fascicular growth similar to fibrosarcoma or monophasic synovial sarcoma. Most often, however, tumors are composed of relatively hypocellular areas alternating with hypercellular areas showing perivascular emphasis, resulting in a mottled appearance at low magnification. The extracellular matrix in less cellular areas is usually myxoid, which can be abundant in up to 10% of cases. Clusters of small rounded blood vessels are commonly seen in high-grade tumors [1].

Histologically, there are some malignant tumors which morphologically resemble MPNST, particularly in the head and neck location, such as spindle cell carcinoma, melanoma or other spindle cell sarcomas, such as monophasic synovial sarcoma or fibrosarcoma [5]. Immunohistochemistry have a great contribution to distinguish between them.

In a MPNST, tumor cells typically express PS100 in a patchy way, and SOX10 in about 50% of cases. EMA and Vimentine can be expressed focally.

Treatment of MPNST is primarily surgical, particularly for small tumors at an early stage of development to achieve complete resection with healthy surgical margins, whereas palliative radiotherapy is recommended when the tumor is at an advanced stage, large size with metastases. The role of adjuvant chemotherapy in the management of MPNST is limited, given its lack of efficacy especially when the tumor is metastatic. [5]

## CONCLUSION

Given their rarity, MPNSTs are tumors relatively difficult to diagnose, especially when it comes to atypical locations such as the nasal cavity.

Sarcomas in general should be taken into account in the differential diagnosis of lesions of the sino-nasal tract. Long-term cures are possible thanks to definitive surgical management followed by one or more adjuvant treatments depending on the grade of the tumor and the condition of the margin.

## CONFLICT OF INTEREST STATEMENT

The authors declare that they have no competing interests.

## FUNDING

No external funding sources are relevant to this submission.

## CONSENT FOR PUBLICATION

Written informed consent for publication of their clinical details and/or clinical images was obtained from the patient.
